# Age-dependent therapeutic effects of liver X receptor-α activation in murine polymicrobial sepsis

**DOI:** 10.1177/1753425915569367

**Published:** 2015-08

**Authors:** Gabriela Botez, Giovanna Piraino, Paul W Hake, John R Ledford, Michael O’Connor, James A Cook, Basilia Zingarelli

**Affiliations:** 1 Division of Critical Care Medicine, Cincinnati Children's Hospital Medical Center, Department of Pediatrics, College of Medicine, University of Cincinnati, Cincinnati, OH, USA; 2 Department of Neurosciences, Medical University of South Carolina, Charleston, SC, USA

**Keywords:** Liver X receptor-α, T0901317, sepsis, lung injury, NF-κB, age, neutrophil infiltration

## Abstract

The severity of sepsis is significantly affected by advanced age; however, age-dependent molecular mechanisms of this susceptibility are unknown. Nuclear liver X receptor-α (LXRα) is a regulator of lipid metabolism with associated anti-inflammatory properties. Here, we investigated the role of LXRα in age-dependent lung injury and outcome of sepsis. Male C57BL/6, LXRα-deficient (LXRα^−/−^) and wild type (WT) (LXRα^+/+^) mice of different ages were subjected to sepsis by cecal ligation and puncture (CLP). In pharmacological studies, treatment with the LXRα ligand T0901317 reduced lung neutrophil infiltration in C57BL/6 mice aged from 1 to 8 mo when compared with vehicle-treated animals subjected to CLP. The LXRα ligand improved survival in young mice (2–3 mo old) but did not affect survival or neutrophil infiltration in mature adult mice (11–13 mo old). Immunoblotting revealed an age-dependent decrease of lung LXRα levels. Young LXRα^−/−^ mice (2–3 mo old) exhibited earlier mortality than age-matched WT mice after CLP. Lung damage and neutrophil infiltration, lung activation of the pro-inflammatory NF-κB and plasma IL-6 levels were higher in LXRα^−/−^ mice 18 h after CLP compared with LXRα^+/+^ mice. This study suggests that the anti-inflammatory properties of LXRα in sepsis are age-dependent and severely compromised in mature adult animals.

## Introduction

Sepsis is a systemic response to infection characterized by hemodynamic and metabolic derangements that may result in septic shock, multiple organ system failure and death. Although it occurs in people of all ages, the incidence and mortality of sepsis is disproportionately increased in elderly adults.^1^ Currently, the treatment of sepsis is centered on general organ supportive measures, ventilator strategies and antibiotic therapy to control the infectious focus. However, these approaches are not always efficacious in reducing the high mortality.^2^ Furthermore age-dependent pathophysiological mechanisms of the systemic inflammatory process, innate immune dysfunction and organ damage are yet to be identified.

The liver X receptor (LXR) is a member of the nuclear receptor superfamily, which is involved in cholesterol, fatty acid and Glc homeostasis, and activated by oxidized forms of cholesterol (oxysterols) and intermediate products of the cholesterol biosynthetic pathway, as well as by synthetic ligands, such as the T0901317.^3–5^ Two LXR subtypes have been identified, LXRα and LXRβ, with different tissue distribution. LXRα is expressed in lung, macrophages, liver, spleen, kidney, adipose tissue and small intestine, whereas LXRβ is ubiquitously expressed.^6,7^ In addition to its cholesterol-regulating properties, LXRα has been reported to control transcriptional programs involved in the inflammatory response. For example, in LPS-stimulated murine macrophages, LXRα activation inhibits the expression of pro-inflammatory cytokines and inducible nitric oxide synthetase.^8,9^ *In vivo* experimental studies have confirmed the anti-inflammatory actions of LXRα in models of LPS-induced lung injury and carrageenan-induced pleurisy.^10,11^ Our laboratory has also demonstrated that treatment of rats with the LXRα synthetic agonist T0901317 reduced plasma cytokines and chemokines, lung neutrophil infiltration and histological damage after hemorrhagic shock.^12^ These *in vitro* and *in vivo* experimental studies have demonstrated that a plausible anti-inflammatory mechanism for LXRα is through inhibition of NF-κB,^8,9^ a transcription factor central to many pro-inflammatory and immune genes involved in sepsis.^13^ However, in these *in vivo* studies the anti-inflammatory effects of LXRα activation have been determined in young rodents only. The ability of LXRα to modulate inflammation in the complex event of an infectious disease setting in aged organisms remains to be determined.

Based on these previous findings, we investigated the biological effects of LXRα modulation on the inflammatory response of polymicrobial sepsis in mice of different ages. Specifically, in *in vivo* pharmacological studies, we demonstrated that treatment with the LXRα ligand T0901317 ameliorated the extent of the sepsis-induced inflammatory response in mice of younger age (1–8 mo old) but not in mature, adult mice (11–13 mo old), which instead exhibited a decrease of the receptor levels in the lung. We also demonstrated that LXRα gene deletion in young septic mice (2–3 mo old) was associated with increased susceptibility to sepsis-induced inflammation, lung injury and earlier mortality. Thus, our data provide evidence that LXRα is an important modulator of innate immune response in sepsis; however, its function declines with age, limiting its therapeutic potentials.

## Materials and methods

### Murine model of polymicrobial sepsis

The investigation conformed to the National Institutes of Health Guide for the Care and Use of Laboratory Animals and commenced with the approval of the Institutional Animal Care and Use Committee. In pharmacological studies, male C57BL/6 mice (Charles River Laboratories, Wilmington MA, USA) were used at an age of between 1 and 1.5 mo (very young group), 2 and 3 mo (young group), 6 and 8 mo (adult group) or between 11 and 13 mo (mature adult group). In complementary studies, male wild type (WT) (LXRα^+/+^) and LXRα-deficient (LXRα^−/−^) mice, both on a mixed-strain background (C57BL/6:129SvEv), were used at an age of between 2 and 3 mo (young group) or between 11 and 13 mo (mature adult group). Polymicrobial sepsis was induced by an established model of cecal ligation and puncture (CLP).^14^ Mice were anesthetized with pentobarbital (40 mg/kg) i.p. After opening the abdomen, the cecum was exteriorized and ligated by a 6-0 silk ligature at its base without obstructing intestinal continuity. The cecum was punctured twice with a 22-G needle and squeezed to excrete small amount of fecal material into the peritoneal cavity. The cecum was then returned into the peritoneal cavity and the abdominal incision was closed with a 6-0 silk ligature suture. After the surgical procedure, mice were resuscitated with 0.6 ml normal saline solution (s.c.) to replace the fluid and blood loss during operation. In pharmacological studies, C57BL/6 mice received vehicle or the LXRα ligand T0901317 (30 mg/kg i.p.) at 1, 6 and 12 h after CLP. In time-course studies, mice (*n* = 4–19) were sacrificed at 3, 6 and 18 h after CLP. Blood and plasma samples, peritoneal fluid, lung, liver and spleen were collected for histological and biochemical studies described below. In survival studies, mice (*n* = 12–20 in each group) were monitored for mortality rates for 72 h.

### Histopathological analysis

Lungs were fixed in 4% paraformaldehyde and embedded in paraffin. Sections were stained with hematoxylin and eosin, and evaluated by five independent observers who were unaware of the experimental protocol. Specifically, lung injury was analyzed by a semiquantitative score based on the following histologic features: (a) alveolar congestion; (b) hemorrhage; (c) infiltration of neutrophils in airspace or vessel wall; and (d) thickness of alveolar wall/hyaline membrane formation. Each feature was graded from 0 to 4 (i.e. absent, minimal, mild, significant, or severe). The four variables were summed to represent the lung injury score (total score, 0–16).

### Myeloperoxidase activity

Myeloperoxidase (MPO) activity was measured as an indicator of neutrophil infiltration in lung tissue after septic shock. Tissues were homogenized in a solution containing 0.5% hexa-decyl-trimethyl-ammonium bromide dissolved in 10 mM potassium phosphate buffer (pH 7.0) and centrifuged for 30 min at 4000 *g* at 4℃. An aliquot of the supernatant was allowed to react with a solution of tetra-methyl-benzidine (1.6 mM) and hydrogen peroxide (0.1 mM). The rate of change in absorbance was measured by spectrophotometry at 650 nm. MPO activity was defined as the quantity of enzyme degrading 1 µmol of hydrogen peroxide/min at 37℃ and expressed in units per 100 mg mass of tissue.

### Cytosol and nuclear protein extraction

Lungs were homogenized using a Polytron homogenizer (Brinkman Instruments, West Orange, NY, USA) in a buffer containing 0.32 M sucrose, 10 mM Tris-HCl (pH 7.4), 1 mM EGTA, 2 mM EDTA, 5 mM NaN_3_, 10 mM β-mercaptoethanol, 20 µM leupeptin, 0.15 µM pepstatin A, 0.2 mM phenylmethanesulfonyl fluoride, 50 mM NaF, 1 mM sodium orthovanadate and 0.4 nM microcystin. Samples were centrifuged at 1000 *g* for 10 min at 4℃ and the supernatants collected as cytosol extracts. The pellets were then solubilized in Triton buffer (1% Triton X-100, 250 mM NaCl, 50 mM Tris HCl at pH 7.5, 3 mM EGTA, 3 mM EDTA, 0.1 mM phenylmethanesulfonyl fluoride, 0.1 mM sodium orthovanadate, 10% glycerol, 2 mM *p*-nitrophenyl phosphate, 0.5% NP-40 and 46 µM aprotinin). The lysates were centrifuged at 15,000 *g* for 30 min at 4℃ and the supernatant collected as nuclear extracts.

### Western blot analysis

Cytosol and nuclear content of LXRα was determined by immunoblot analysis on nitrocellulose membranes using primary Abs against LXRα and secondary peroxidase-conjugated Ab. Membranes were also re-probed with primary Ab against β-actin to ensure equal loading samples. Immunoreaction was visualized by chemiluminescence. Densitometric analysis of blots was performed using Quantity One (Bio-Rad Laboratories, Des Plaines, IL, USA).

### Measurement of NF-κB DNA binding activity

NF-κB DNA binding activity was analyzed by a TransAM Transcription Factor assay kit specific for the activated form of p65 (Active Motif, Carlsbad, CA, USA) using the NF-κB consensus site (5′–GGGACTTTCC-3′) according to the manufacturer’s protocol.

### Bacterial colony counts

Bacterial clearance was indirectly assessed by counting bacterial colonies in blood and peritoneal fluid, and homogenized samples of spleen, liver and lung at 18 h after CLP surgery. Serial dilutions of samples were cultured on 5% sheep blood trypticase soy agar plates (BBL Stacker Plate TSA II, BD Biosciences, Sparks, MD, USA) and incubated at 37℃ in aerobic conditions for 24 h. Colony forming units (CFU) were counted and log-transformed to obtain a normal distribution. Results were expressed as log CFU/ml for blood, log CFU/g for tissues and log CFU/ mouse for peritoneal lavage fluid.

### Plasma levels of cytokines and adipokines

Plasma levels of IL-6 and TNF-α were evaluated by a commercially available solid-phase ELISA kit (R&D Systems, Minneapolis, MN, USA). Plasma levels of resistin and leptin were evaluated by a commercially available multiplex array system (Linco-Research, St. Charles, MO, USA) using the protocols recommended by the manufacturer.

### Materials

The LXRα agonist T0901317 was obtained from Cayman Chemical (Ann Arbor, MI, USA). The primary Ab directed at LXRα was obtained from Abcam (Cambridge, MA, USA). The secondary Abs and β-actin were obtained from Santa Cruz Biotechnology (Santa Cruz, CA, USA). All other chemicals were obtained from Sigma-Aldrich (St. Louis, MO, USA).

### Data analysis

Statistical analysis was performed using SigmaStat for Windows Version 3.10 (SysStat Software, San Jose, CA, USA). Data in the figures and text are expressed as means ± SEM or median with 25th and 75th percentiles of *n* observations (*n* = 4–19 animals for each group). The results were examined by ANOVA followed by the Student–Newman–Keuls’s correction post hoc *t*-test. Statistical analysis of damage scores was performed using the Mann-–Whitney Rank Sum test; when normality and equal variance passed, data were further analyzed by *t*-test. The Gehan–Breslow and log-rank tests were used to compare differences in survival rates. A value of *P* < 0.05 was considered significant.

## Results

### Pharmacological activation of LXRα improves survival in young but not mature adult mice

**Figure 1.**
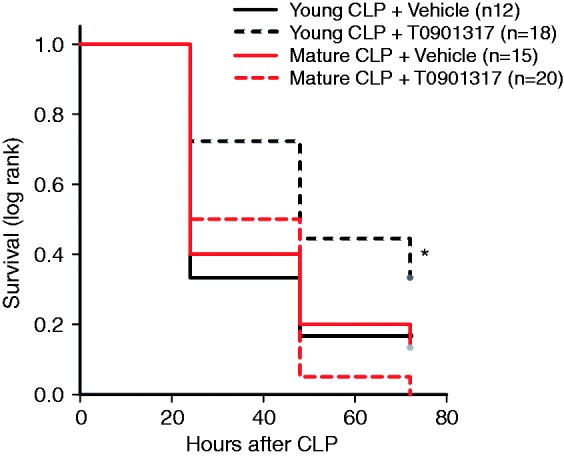
Kaplan–Meier curve of survival monitored for 72 h. *P = 0.049 by Gehan–Breslow analysis. Male C57BL/6 mice received T0901317 (30 mg/kg i. p.) or vehicle at 1, 6 and 12 h following CLP.

In order to achieve a similar magnitude of organ inflammatory response and mortality between different groups of age and to evaluate the potential therapeutic efficacy of LXRα activation, we used a severe model of CLP (22-G needle) without antibiotics to produce high mortality during the early phase. With this procedure, the onset of clinical symptoms in C57BL/6 mice, such as piloerection and inactivity, were observed by 6–12 h. When survival curves were monitored for 3 d after CLP, mortality rates were similar between vehicle-treated young (2–3 mo old) and mature adult (11–13 mo old) groups (Figure 1). Treatment with the LXRα ligand T0901317 improved survival in young mice (*P* = 0.049); however, it did not modify survival rate in mature adult mice when compared with age-matched vehicle-treated mice (*P* = 0.941) (Figure 1).

### Age-dependent effect of pharmacological activation of LXRα in sepsis-induced lung injury

**Figure 2.**
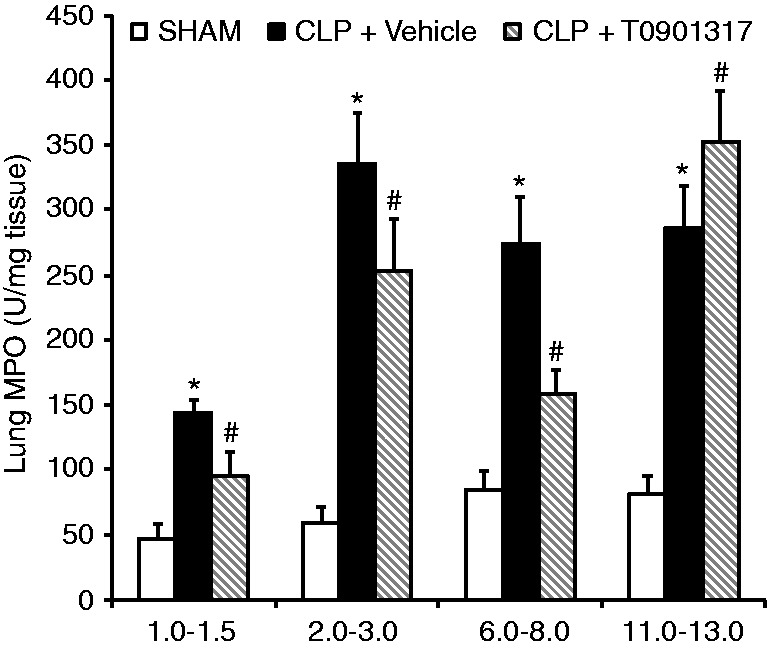
MPO activity in lung at 6 h after CLP. Data are means ± SEM of 5–18 mice for each group. *P < 0.05 vs, age-matched sham mice; #*P* < 0.05 vs. vehicle treatment of age-matched group. Mice received T0901317 (30 mg/kg i.p.) or vehicle at 1 h following CLP.

As a serious consequence of sepsis is the occurrence of multiple organ failure, which is preceded by accumulation of neutrophils in major vital organs, we evaluated the effect of the LXRα ligand T0901317 on sepsis-induced lung neutrophil infiltration by measurement of the activity of MPO, an enzyme specific to neutrophil lysosomes. Treatment with T0901317 (30 mg/kg i.p.) reduced MPO activity in different age groups of mice (from 1 to 8 mo) compared with their age-matched vehicle-treated mice subjected to CLP (Figure 2). On the contrary, treatment with T0901317 worsened neutrophil infiltration in mature adult mice (11–13 mo) when compared with age-matched vehicle-treated mice, thus suggesting the potential of non-specific toxic effects (Figure 2).

### Age-dependent down-regulation of LXRα in the lung

**Figure 3.**
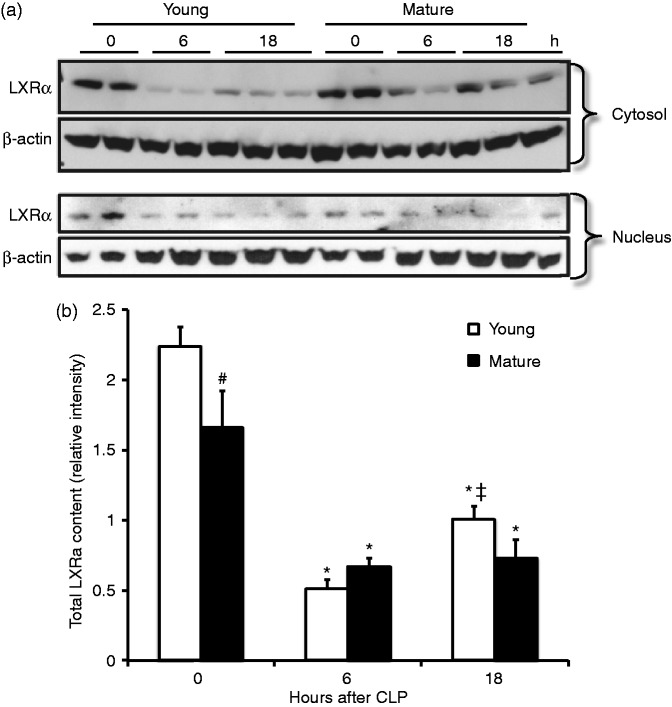
(a) Western blot of cytosol and nuclear LXRα content and β-actin (used as loading control protein) in the lung at 0, 6 and 18 h after CLP. (b) Image analyses of LXRα content as determined by densitometry. Data are mean ± SEM of 4–12 animals for each group and are expressed as relative intensity units. *P < 0.05 vs. age-matched controls; #*P* < 0.05 vs. the young group; ‡ *P* < 0.05 vs. 6 h time point of age matched-mice.

As pharmacological activation of LXRα afforded protection in young and adult animals only, but not in the mature adult groups, we investigated whether sepsis was associated with age-dependent changes in LXRα levels. By Western blot analysis, we observed that, when compared with basal levels of age-matched sham animals, cytosol and nuclear content of LXRα in lungs was reduced at 6 h after CLP in both young and mature adult groups. In sham mature adult mice, however, total levels of protein were lower when compared with the young group (Figure 3). At 18 h after CLP, there was a partial replenishment of this receptor in both young and mature adult mice; however, total levels of LXRα were lower, although not statistically different, in mature adult groups when compared with young mice. In addition, mature adult mice exhibited retention of the receptor in the cytosol when compared with young mice (Figure 3).

### Genetic ablation of LXRα exacerbates early mortality rate in young mice

**Figure 4.**
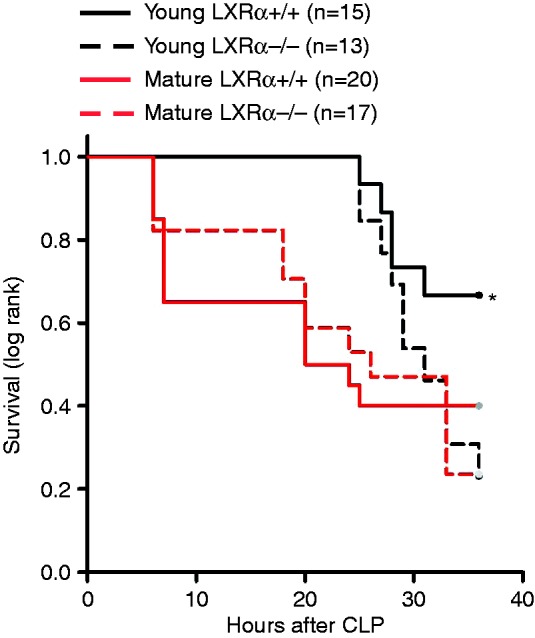
Kaplan–Meier curve of survival monitored for 36 h. *P = 0.049 by log-rank analysis vs. young LXRα^–/–^, mature adult LXRα^+/+^ and mature adult LXRα^–/–^.

To confirm the protective role of LXRα in sepsis, we performed additional survival studies in genetically LXRα-deficient mice and their WT controls. When analysis of survival rate was focused only on the initial period of sepsis, we observed that death occurred earlier in young LXRα^−/−^ mice and in both mature adult LXRα^+/+^ and mature adult LXRα^−/−^ mice when compared with young LXRα^+/+^ WT animals (*P* = 0.044 at 36 h after CLP) (Figure 4). However, at 72 h after CLP, mortality rates (approximately 90–100%) were similar between the young (2–3 mo old) and mature adult (11–13 mo old) groups of both genotypes (data not shown).

### Genetic ablation of LXRα exacerbates lung injury in young mice

**Figure 5.**
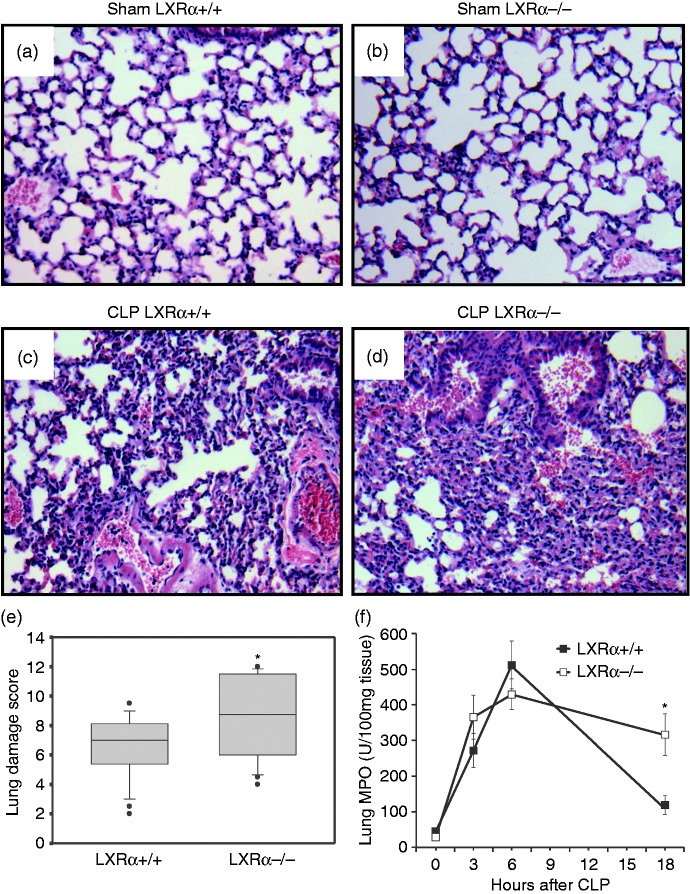
Representative histology photomicrographs of lung sections of WT LXRα^+/+^ and LXRα^–/–^ mice subjected to polymicrobial sepsis. Lungs were harvested at 18 h after CLP and were stained with hematoxylin and eosin. Normal lung architecture in (a, b) sham mice of both genotypes and (c, d) lung derangement in septic mice at 18 h after CLP (c, d). Magnification × 100. A similar pattern was seen in 4–6 different tissue sections in each experimental group. (e) Histopathological scores of lung sections (*n* = 5 mice for each group). Lung injury was scored from 0 (no damage) to 16 (maximum damage). Box plots represent 25th percentile, median and 75th percentile; error bars define 10th and 90th percentiles. (f) Time-course analysis of lung MPO activity after CLP in WT LXRα^+/+^ mice and LXRα^–/–^ mice. Data represent the mean ± SEM of 4–9 animals per group. *P < 0.05 vs. LXRα^+/+^ mice.

Having determined that LXRα ablation could reduce short-term survival, we focused on organ injury changes. Given the early high mortality observed in this model of CLP, examination was limited to early time points of CLP in the young group only. In the lung of WT mice, histological examination revealed extravasation of red cells and accumulation of neutrophils into the air spaces at 18 h after CLP (Figure 5). In LXRα^−/−^ mice, lung injury was more severe and consisted of reduced alveolar air spaces, large hemorrhagic areas and infiltration of inflammatory cells. We also measured activity of MPO to confirm tissue neutrophil infiltration. Lung neutrophil infiltration was similar in the two genotypes in the earlier phases of sepsis at 3 and 6 h after CLP. However, neutrophil infiltration was higher in LXRα^−/−^ mice when compared with WT mice at 18 h after CLP, probably signifying an inability to turn off the inflammatory process (Figure 5f). To examine the effect of genetic absence of LXRα on the capability to eliminate bacterial pathogens, we counted bacterial CFU in blood, peritoneal fluid, lung, spleen and liver at 18 h after CLP. The numbers of bacterial colonies were similar in all these compartments in LXRα^+/+^ and LXRα^−/−^ mice (Table 1).

**Table 1.** Tissue bacterial content in young LXRα^+/+^ and LXRα^–/–^ mice.

Tissues	LXRα^+/+^ mice CFU (log)	LXRα^–/–^ mice CFU (log)
Blood	3.48 ± 0.34	3.08 ± 0.44
Peritoneal fluid	4.73 ± 0.47	4.46 ± 0.46
Lung	2.31 ± 0.26	2.11 ± 0.28
Spleen	2.96 ± 0.29	2.55 ± 0.23
Liver	2.18 ± 0.25	1.95 ± 0.26

Tissue samples were harvested at 18 h after CLP surgery. CFU were counted and log-transformed to obtain a normal distribution. Data ± SEM (*n* = 12 mice for each group) were expressed as log CFU/ml for blood, log CFU/mouse for peritoneal lavage fluid and log CFU/g for tissues.

### Genetic ablation of LXRα increases systemic elevation of IL-6 in young mice

**Figure 6.**
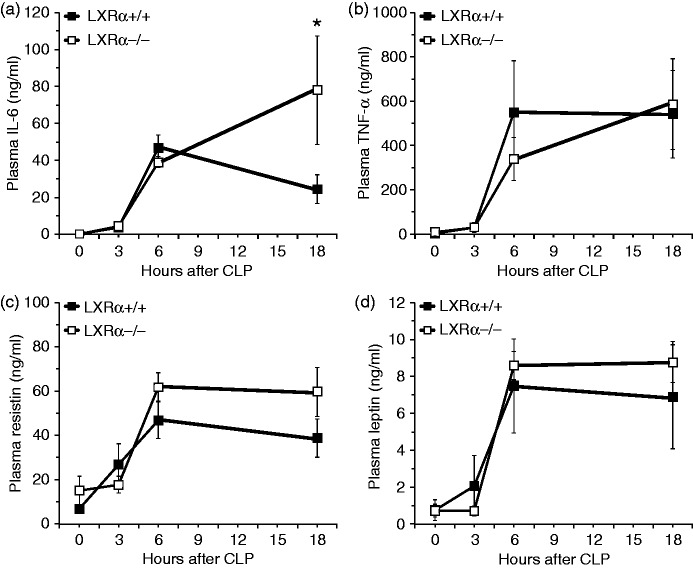
Plasma levels of (a) IL-6, (b) TNF-α, (c) resistin and (d) leptin WT LXRα^+/+^ and LXRα^–/–^ mice at 0, 6 and 18 h after CLP. Each data point represents the mean ± SEM of 4–6 animals for each group. *P < 0.05 vs. –WT mice at the same time point.

Increased early mortality of LXRα^−/−^ mice was associated with higher plasma levels of the pro-inflammatory IL-6 when compared with WT mice at 18 h after CLP. Plasma levels of other pro-inflammatory cytokines and adipokines, such as TNF-α, resistin and leptin, were similarly elevated in both genotypes (Figure 6).

### Decrease of LXRα content is associated with up-regulation of NF-κB activation in the lung following polymicrobial sepsis

**Figure 7.**
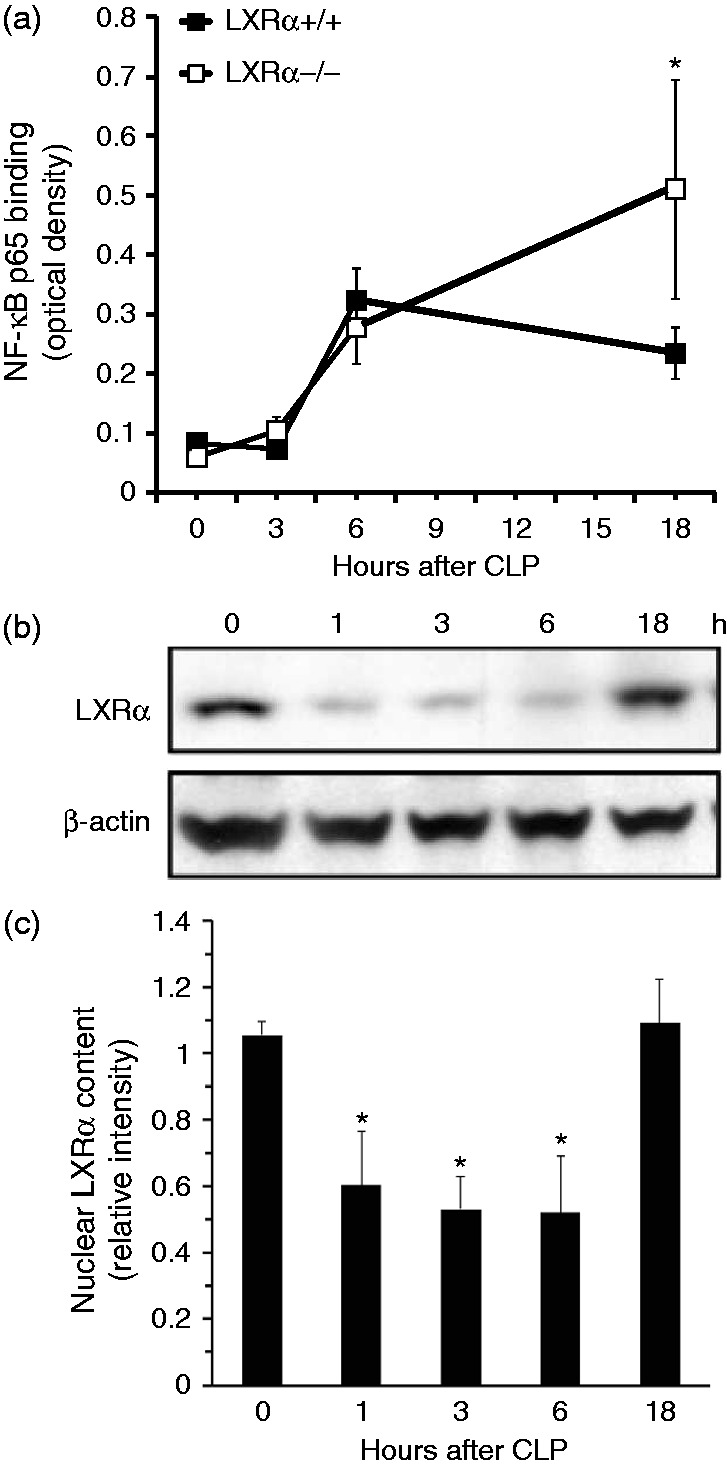
Sepsis induces up-regulation of NF-κB activity and decrease of LXRα content in the lung. (a) Activity of the p65 subunit of NF-κB in lung nuclear extracts at 0, 3, 6 and 18 h after CLP. Data are mean ± SEM of 4–6 animals for each group and are expressed as optical density units. *P < 0.05 vs. LXRα^+/+^ mice. (b) Western blot analysis of LXRα and β-actin (used as loading control protein) in lung nuclear extracts. (c) Image analyses of LXRα content as determined by densitometry. Data are mean ± SEM of 4–6 animals for each group and are expressed as relative intensity units. *P < 0.05 vs. time 0.

To determine the inflammatory signaling pathways affected by LXRα genetic deletion, we also investigated the kinetics of NF-κB activation. NF-κB activity was similar in the two genotypes in the earlier phases of sepsis. However, NF-κB activity persisted at higher levels in LXRα^−/−^ mice when compared with WT mice at 18 h after CLP (Figure 7a). Interestingly, by Western blot analysis, we observed that the nuclear content of LXRα decreased in a time-dependent manner up to 6 h after CLP and was then replenished at 18 h in lung nuclear extracts of WT LXRα^+/+^ mice (Figures 7b, c). These data are consistent with the lung injury pattern, which was different in the two genotypes at 18 h.

## Discussion

Our studies demonstrate that pharmacological activation of LXRα by T0901317 reduces pulmonary neutrophil infiltration and improves survival in young mice subjected to polymicrobial sepsis. On the contrary, our studies demonstrate that mature adult mice have less total LXRα, thus losing their capability to respond to the protective effects of the LXRα ligand. Thus, our data suggest that LXRα is an important regulator of the innate immune response; however, a decrease of LXRα content in the lung may represent an additional mechanism underlying the age-dependent poor recovery in sepsis.

LXRα is a nuclear receptor with widely recognized roles in cholesterol metabolism and inflammation. In the lung, the receptor is expressed in macrophages, neutrophils, type II pneumocytes and airway smooth muscle cells.^7,15,16^ Therefore, it is biologically plausible that LXRα may play a part in the delicate pro- vs. anti-inflammatory balance. Previous work has already shown that activation of LXRα attenuates inflammation in a variety of preclinical models of lung injury in young rodents, such as LPS administration,^10,17^ pneumonia,^15^ carrageenan-induced pleurisy^11^ and hemorrhagic shock.^12^

Acute lung injury involves intrapulmonary neutrophil accumulation and activation with the release of cytotoxic compounds, which leads to loss of the alveolar epithelial and capillary endothelial barrier function and increase in permeability of the alveolo-capillary membrane.^18,19^ In the present study, we used complementary pharmacological gain-of-function and genetic loss-of-function approaches to demonstrate that LXRα signaling is instrumental in modulating neutrophil infiltration in sepsis-induced lung injury. Treatment of C57BL/6 mice (1–8 mo old) with the LXRα ligand T0901317 significantly reduced pulmonary tissue MPO activity compared with the vehicle-treated group. Conversely, in young LXRα-deficient animals (2–3 mo old), the degree of lung neutrophil infiltration was significantly higher than in the lungs of WT animals at a late time after sepsis and was associated with worse histological damage. These findings are consistent with our previous work demonstrating that LXRα activation leads to a reduction in lung neutrophil infiltration in a model of hemorrhagic shock,^12^ as well as the work of others using models of LPS-induced lung injury,^10^ pneumonia^15^ and carrageenan-induced pleurisy.^11^

Early therapeutic intervention is critical for a positive outcome in septic patients.^20^ However, the early pathophysiological events of organ damage have not been yet defined. Given the dynamic nature of the inflammatory cascade that leads to sepsis-induced lung injury, we determined the kinetics of LXRα levels after the induction of sepsis. We found that lung content of LXRα in LXRα^+/+^ mice was significantly lower at the early time points after CLP compared with basal levels and it partially recovered at 18 h later. Of more importance, the temporal partial recovery of LXRα nuclear content in LXRα^+/+^ mice was associated with lower pulmonary leukocyte recruitment, less lung damage and lower levels of the pro-inflammatory cytokine IL-6 when compared with septic LXRα^−/−^ mice. Interestingly, plasma levels of TNF-α, resistin and leptin were not different between the two genotypes. Thus, deficiency of LXRα^−/−^ seems to negatively affect the outcome of septic animals, probably by enhancing the pro-inflammatory cytokine IL-6 and lung neutrophil infiltration, while not affecting the production of the adipokines resistin and leptin. Other studies confirm that LXRα is an important contributor of the protective immune response during infections by modulating the cytokine response.^21^ Furthermore, LXRα activation has been shown to potently inhibit the release of IL-6 and the development of experimental skin fibrosis in mice.^22^ Taken together with these previous reports, our data suggest that LXRα is a part of the early regulatory host response to CLP-induced sepsis and may be used as a therapeutic target; its initial decrease may favor pro-inflammatory mechanisms, while its subsequent replenishment may promote anti-inflammatory mechanisms, as the body attempts to limit potentially damaging inflammation.

Our results are consistent with other studies, which have shown that the content of LXRα decreases after LPS-induced lung injury.^10^ Interestingly, a similar dynamic of LXRα levels was observed in the livers of mice subjected to CLP,^23^ supporting the notion that LXRα might be involved in regulating sepsis-related inflammation in a variety of organs. Our results are also consistent with our previous reports demonstrating that the acute phase response of septic shock, or endotoxemia, is associated with a marked decrease of other nuclear hormone receptors of the peroxisome proliferator-activated receptor (PPAR) family, such as PPARα, PPARγ and PPARδ, which share with LXRα important regulatory functions in lipid and Glc metabolism.^24–26^ Experimental *in vitro* studies further support that the inflammatory response induced by pro-inflammatory cytokines can directly induce changes in the levels of metabolic nuclear receptors including PPARα, PPARγ and LXRα in Hep3B human hepatoma cells.^27^ Other studies have also demonstrated that aging is associated with reduced activity of several nuclear receptors, including LXRα, in rat brain.^28^ Although the mechanisms of protein loss have not yet determined, it is possible that post-translational modifications of the nuclear receptor may influence the protein stability and its dynamic changes. In fact, it has been previously demonstrated that LXRα deacetylation favors its ubiquitination and subsequent degradation, whereas specific LXRα ligands prevent protein degradation and increase the recruitment of the receptor to the target gene promoters.^29,30^

In the present study, young LXRα-deficient mice exhibited significantly higher NF-κB activity in the lung at 18 h after CLP compared with LXRα^+/+^ mice, further supporting the notion that LXRα may have a regulatory role on the pro-inflammatory pathways. The NF-κB is a nuclear transcription factor that plays an important role in the coordination of innate and adaptive immune responses in sepsis by modulating the expression of many pro-inflammatory genes.^13^ There is evidence to suggest that at least some of the anti-inflammatory effects of LXRα are mediated through inhibition of the NF-κB pathway.^31,32^ Our laboratory has also previously demonstrated that LXRα activation inhibits the LPS-induced inflammatory response in murine macrophages by inhibiting the NF-κB pathway.^9^ Moreover, we have shown that activation of LXRα inhibits NF-κB binding to DNA in the lung in a rodent model of hemorrhagic shock and resuscitation.^12^

Our CLP procedure without fluid resuscitation or antibiotic treatment resulted in a severe septic shock, with 90–100% mortality at 3 d after surgery. Nevertheless, despite the rapid demise, the presence of a functional LXRα in young WT LXRα^+/+^ mice was associated with delayed onset of mortality when compared with young LXRα^−/−^ mice. Furthermore, even in this severe model of sepsis, treatment of young septic C57BL/6 mice with the LXRα ligand T0901317 significantly improved survival compared with vehicle treatment. Thus, our data suggest that an adequate LXRα receptor reserve offers a therapeutic target for the use of specific ligands in young mice.

Age itself is an independent risk factor for death in patients with severe sepsis.^1,33^ In support of the clinical observation, several experimental studies have demonstrated that aged animals experience increased severity of the inflammatory response and mortality when compared with younger animals.^34–37^ In our pharmacological studies, in order to investigate the therapeutic efficacy of LXRα activation, we calibrated injury severity in the CLP procedure to produce similar mortality in young and mature adult mice, thus avoiding the potential mortality disadvantage of aged animals. We demonstrated that post-treatment with the LXRα ligand T0901317 reduced lung neutrophil infiltration and improved survival after sepsis in young mice when compared with vehicle treatment; however, it failed to provide any protective effects in mature adult mice. At molecular analysis, mature adult mice had a significant lower content of LXRα in the lung when compared with young animals. Therefore, it is plausible that the lack of therapeutic effect of the ligand T0901317 in mature adult mice may be secondary to the poor availability of total receptor for ligand engagement. The age-dependent reduced content of the receptor may also explain the fact that mature adult WT LXRα^+/+^ mice had similar outcomes as the mature adult LXRα^−/−^ mice after sepsis.

In conclusion, our study shows that a reduction of LXRα protein is associated with lung injury after sepsis. Young, but not mature adult mice, have an adequate receptor reserve, which may be a valid therapeutic target for sepsis treatment. It is clear, therefore, that the decrease of LXRα levels with advanced age limits the therapeutic potentials of ligands for this receptor. The molecular mechanisms that mediate the age-dependent decrease of LXRα remain to be elucidated.
